# Effect of adding dexmedetomidine as an adjuvant to bupivacaine in ultrasound-guided erector spinae plane block for postoperative pain management following modified radical mastectomy: a randomized controlled trial

**DOI:** 10.1186/s12893-026-03557-0

**Published:** 2026-03-04

**Authors:** Abanoub Mouris Feltaoos, Hatem Said Abdelhamed, Karim Youssef Kamal, Ashraf Elsayed Elagamy, Ahmed Mostafa Abdulmageed

**Affiliations:** Department of Anesthesia, Intensive Care and Pain Management, Faculty of Medicine, Ain Shams University, Abbasia, Cairo, Egypt

**Keywords:** Erector spinae plane block, Modified radical mastectomy, Dexmedetomidine, Bupivacaine

## Abstract

**Background:**

Postoperative pain after modified radical mastectomy (MRM) can lead to significant morbidity. The erector spinae plane block (ESPB) has emerged as an effective regional anesthesia technique. This study evaluated the effect of adding dexmedetomidine to bupivacaine on postoperative analgesia following ESPB in MRM.

**Patients and methods:**

This double-blinded, randomized controlled trial included 60 female patients (ASA I–II, age 30–65 years, BMI < 35 kg/m²) undergoing MRM at Ain Shams University Hospitals between August 2023 and July 2024. Patients were randomly allocated into two groups (*n* = 30 each): Group B received unilateral ESPB with 20 ml of 0.5% bupivacaine, and Group BD received 10 ml of 0.5% bupivacaine plus dexmedetomidine (100 µg/mL, 1 µg/kg) diluted to a total volume of 20 ml with 0.9% saline. Blocks were performed postoperatively in lateral decubitus position under ultrasound guidance. Rescue analgesia consisted of IV nalbuphine 5 mg for VAS > 4.

**Results:**

The median (IQR) time for request of the initial dose rescue analgesia was significantly delayed in group BD compared to group B [690 (525–795) versus 360 (300–420), respectively, P-value < 0.001]. The number of patients that required nalbuphine was significantly lower in group BD compared to group B (P-value = 0.004). The median (IQR) visual analogue scale (VAS) scores at 6 h postoperative were significantly reduced in group BD compared to group B [1 (1–2) versus 4 (4–4), P-value < 0.001].

**Conclusion:**

Our study’s results indicated that adding dexmedetomidine to bupivacaine in ESPB enhanced its efficacy, extended postoperative pain relief, and reduced the need for nalbuphine.

**Clinical Trial registration:**

The identification code in the clinicaltrials.gov database is NCT06022614, registration date: 1 September 2023, and it is also listed in the Pan African Clinical Trials Registry with the ID PACTR202307885435649, registration date: 27 July 2023. The research adhered to the CONSORT 20205 framework.

Randomized controlled trial registered on 1 September 2023.

**Supplementary Information:**

The online version contains supplementary material available at 10.1186/s12893-026-03557-0.

## Introduction

Breast cancer is one of the most prevalent diseases affecting women, which can affect one in eight women at some point in their lives. Every year, it is responsible for over 10 of all novel cancer diagnoses. A commonly performed surgical method for treating breast cancer is the modified radical mastectomy. This kind of surgery requires appropriate pain control due to its association with intense post-surgical pain and the potential development of post-mastectomy syndrome, which is a chronic pain condition that can develop after breast surgery [1].

It is characterized by persistent pain in the chest, armpit, and/or arm, often described as neuropathic pain, and occurs more frequently in younger women. Those who have undergone axillary lymph node dissection and those who have received radiation therapy may be at increased risk [2].

Appropriate pain management is important because this persistent pain condition affects 25% to 60% of patients after breast surgery and can last for months. As a consequence of that, numerous regional nerve blocks, for instance, paravertebral blocks, have been suggested as an analgesic type for breast surgery [3].

The erector spinae plane block (ESPB) was described in 2016 by Forero et al. [4]. as a new interfascial plane block designed to relieve thoracic neuropathic pain. Subsequent research has indicated that ESPB can facilitate efficient pain management in breast surgery procedures. ESPB can be accomplished as part of multimodal analgesia by administering local anesthetics to the plane in between the erector spinae muscle and thoracic transverse process. The erector spinae muscles are structurally along the thoracoabdominal fascia, so extensive craniocaudal spread may be achieved with ESPB [5].

Several additives were mixed with local anesthetics to improve the quality and lengthen the period of the peripheral nerve blocks’ effect. Dexmedetomidine is a highly selective α-2 adrenoceptor agonist that is tenfold more selective than clonidine; it has sedative, analgesic, sympatholytic, and anxiolytic effects. It can also lengthen and speed up the onset of nerve blocks when used with LA. The mechanisms of dexmedetomidine administration in the peripheral nerve block are as follows: it inhibits the function of sodium channels and neuronal potassium current and blocks the hyperpolarization-activated cyclic nucleotide-gated channels, leading to the inhibition of substance P release in the nociceptive pathway at the dorsal root neuron.It may also be helpful in postoperative cancer surgery because of its antiemetic qualities, which are linked to a lower incidence of postoperative nausea and vomiting [6, 7].

Bupivacaine is a powerful amide local anesthetic that numbs specific body areas by blocking sodium channels in nerve cells, stopping pain signals from reaching the brain, causing reversible loss of feeling, and it works by preventing nerve depolarization, leading to a prolonged effect due to its high lipid solubility [8]. Therefore, this study aimed to evaluate effect of adding dexmedetomidine as an adjuvant to bupivacaine in ultrasound-guided ESPB for post-modified radical mastectomy pain management.

## Patients and methods

**Fig. 1:**
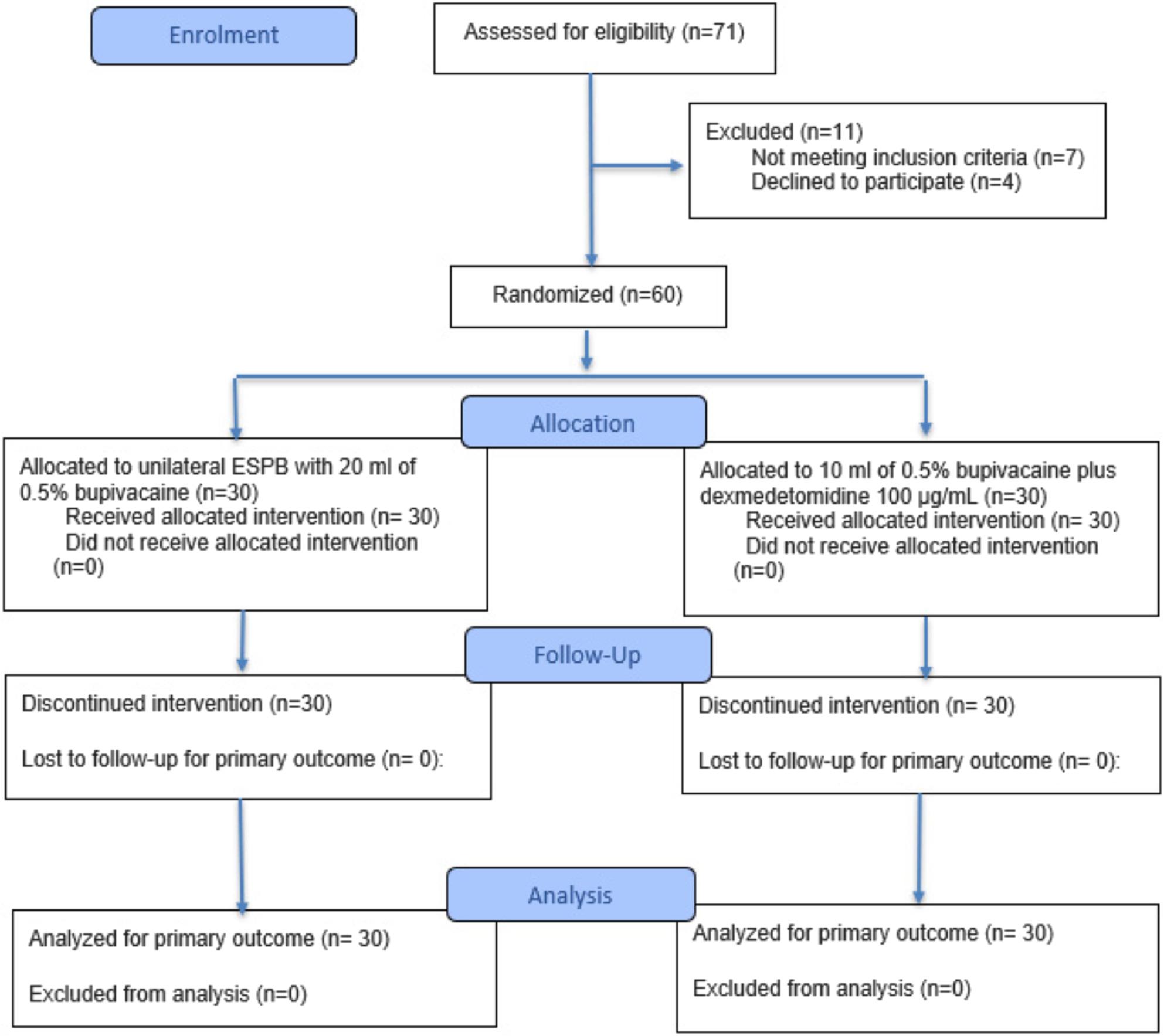
Consort flow diagram

This prospective, double -blind, randomized controlled trial was conducted from August 2023 to July 2024 at Ain Shams University Hospitals after approval from the Research Ethics Committee (FMASU MD145/2023). The trial was registered at ClinicalTrials.gov (NCT06022614) and the Pan African Clinical Trials Registry (PACTR202307885435649). All participants provided written informed consent. The study adhered to CONSORT 2025 guidelines, with the flow diagram included as Fig. 1.

Eligible participants were female patients aged 30–65 years, ASA physical status I–II, with BMI < 35 kg/m², scheduled for MRM. Exclusion criteria included hypersensitivity to study drugs, metastatic breast cancer, psychiatric or neurological disorders affecting pain perception (assessed using a preoperative psychiatric interview and patient history), coagulopathy, local infection at the block site, chronic opioid use, preexisting chronic pain, or prior breast surgery. Patients who declined participation were not enrolled.

Randomization was performed using a computer-generated sequence (http://www.randomizer.org) by an independent investigator. Sequentially numbered, opaque, sealed envelopes were prepared, and the group allocation was concealed until the time of block placement. A nurse, not involved in outcome assessment, opened the envelope and administered the block according to the allocation. This procedure ensures patient blinding, though outcome assessors were also blinded to minimize detection bias.

Group B received unilateral ESPB with 20 ml of 0.5% bupivacaine. Group BD received 10 ml of 0.5% bupivacaine plus dexmedetomidine 100 µg/mL, 1 µg/kg, diluted with 0.9% saline to a total volume of 20 ml. The volume of dexmedetomidine was adjusted per patient weight to deliver 1 µg/kg, then made up to a total of 1 ml from the stock solution. This ensured consistent total injectate volume across groups while maintaining the correct weight-adjusted dose.

Blocks were performed postoperatively with patients in lateral decubitus position on the surgical side, using a high-frequency linear probe (6–13 MHz) of the SonoSite M-Turbo C^®^ ultrasound machine. After aseptic preparation, a 22G, 10-cm nerve block needle was advanced in-plane to contact the transverse process. After negative aspiration, the study solution was injected, and spread was confirmed by real-time ultrasound visualization.

General anesthesia was induced with IV fentanyl 2 µg/kg, propofol 2 mg/kg, and atracurium 0.5 mg/kg, followed by endotracheal intubation. Anesthesia was maintained with isoflurane (MAC 1.2%) and additional atracurium (0.1 mg/kg) as required. Neuromuscular blockade was reversed with neostigmine 0.05 mg/kg and atropine 0.02 mg/kg before extubation.

Postoperatively, patients were monitored in the PACU, and standard parameters (HR, MAP, SpO₂) were recorded at 0, 1, 2, 6, 12, and 24 h. Pain was assessed using the visual analog scale (VAS, 0–10), and IV paracetamol 1 g every 8 h was provided. Rescue analgesia (IV nalbuphine 5 mg) was given on-demand for VAS > 4, with a maximum allowable dose of 0.15 mg/kg/day.

The adverse effects were assessed: hypotension (decrease in basal mean arterial blood pressure by 20%) was treated with I.V. fluid, bradycardia (defined by decrease in basal heart rate less than 60 beats/min) was treated by IV atropine 0.02 mg/kg, respiratory depression (the SpO_2_ < 95% and need O_2_ supplementation), and postoperative nausea and vomiting (PONV) was treated by IV ondansetron 0.1 mg/kg.

### Primary and secondary outcomes

Primary outcome: time to first request for rescue analgesia. Secondary outcomes: VAS scores at predefined intervals and total nalbuphine consumption in the first 24 h.

### Sample size calculation

The sample size calculation was performed using G. power 3.1.9.2 (Universitat Kiel, Germany). The sample size was calculated based on the following considerations: 0.05 α error, 0.804 effect size, and 80% power of the study to demonstrate a 10% increase in the time to first request for rescue analgesia with Group BD than Group B (mean 89 and SD 8.3 according to a previous study [9]). Four cases were added to each group to overcome dropout. Therefore, 30 patients were allocated in each group.

### Statistical analysis

Statistical analysis was done by SPSS v29 (IBM Inc., Chicago, IL, USA). Shapiro-Wilks test and histograms were used to evaluate the normality of the data distribution. Quantitative parametric data were presented as mean and standard deviation (SD) and compared between the two groups utilizing unpaired Student’s t-test. Quantitative non-parametric data were presented as median and interquartile range (IQR) and compared between the two groups utilizing Mann Whitney test. Qualitative variable data were presented as frequency (%) and analyzed using the Chi-square or Fisher’s exact test when appropriate. A two-tailed P-value < 0.05 was considered statistically significant.

## Results

71 patients were assessed for eligibility, seven patients did not meet the criteria, and four patients refused to participate in the study. The remaining patients were randomly allocated into two equal groups (30 patients in each). All allocated patients were followed up and analyzed statistically as recorded in Fig. 1.

### Demographic data

Regarding demographic information of the cases under study (age, body mass index (BMI), American society of anesthesiologists (ASA) score, diabetes mellitus, hypertension, tumor stage and duration of surgery), there was no statistically significant distinction between groups B and BD (*P*-value > 0.05). Table 1.

**Table 1:** Comparison of demographic data and duration of surgery using unpaired student's t-test, Fisher exact and Chi-square between the two studied groups

		Group B(*n*=30)	Group BD(*n*=30)	*p*-value	MD or RR (95% CI)
Age (years)		50.8 ±3.67	51.93 ±3.70	0.239^t^	1.13 (-0.78: 3.04)
BMI (kg/m^2^)		25.83 ±2.61	26.50 ±3.37	0.393^t^	0.67 (-0.89: 2.23)
ASA	I	13(43.3%)	11(36.7%)	0.598^X2^	1.13 (-0.78 3.04)
	II	17(56.7%)	19(63.3%)		
Comorbidities	Diabetes mellitus	3 (10%)	2 (6.67%)	1^FE^	1.5(0.27:8.34)
	Hypertension	7 (23.33%)	10 (33.33%)	0.390^X2^	0.7(0.31:1.59)
Tumor stage	Stage I	10 (33.33%)	12 (40%)	0.866	---
	Stage II	11 (36.67%)	10 (33.33%)		
	Stage III	9 (30%)	8 (26.67%)		
Duration of surgery (mins)		136.67 ±19.54	139.33 ±20.83	0.611^t^	2.66 (-7.78: 13.09)

Data are presented as mean ±SD or frequency (%), *BMI* Body mass index, *ASA* American society of anesthesiologists, *t* Unpaired student's t-test, *FE* Fisher exact, *X*^2^ Chi-square, *MD* Mean difference, *RR* Relative risk, *CI* Confidence interval

### Hemodynamics

**Fig. 2:**
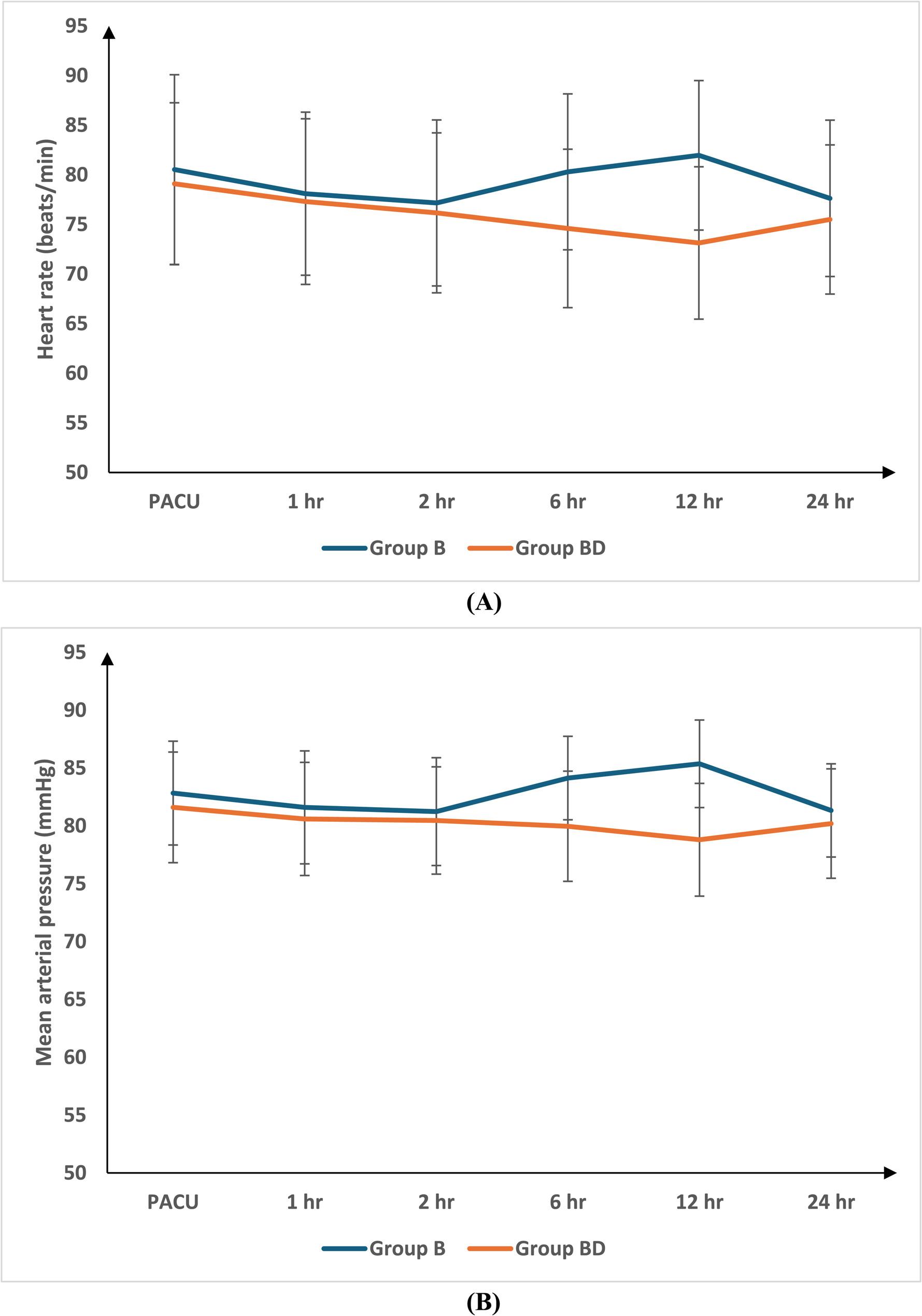
**A** Heart rate (**B**) Mean arterial pressure of the studied group

Both groups were assessed regarding heart rate (HR) and mean arterial pressure (MAP) at the post-anesthesia care unit (PACU), 1, 2, 6, 12, and 24 h postoperatively. Group BD showed a more significant decrease at 6 and 12 h after surgery in HR and MAP than group B (P-value < 0.05), no notable statistical difference in hemodynamics was recorded between the two groups at any other time point, as recorded in (Fig.2).

### Pain control

The two groups were compared regarding time for the initial dose of rescue analgesia, visual analogue score (VAS score), and overall nalbuphine usage through the postoperative period.

The findings also indicated that the average time until the initial request for rescue analgesia was notably longer in group BD relative to group B, and this distinction was statistically notable (p-value < 0.001). By the conclusion of the first 24 h after surgery, seventeen cases in group B needed rescue analgesics, while only 6 cases in group BD needed rescue analgesics; number of patients in group BD had consumed nalbuphine was statistically significantly less than number of patients in group B (p-value = 0.004). The amount of nalbuphine consumption was lower in group BD than group B with insignificant difference (P-value = 0.082) as shown in Table 2.

**Table 2:** Comparison of time for request of the initial dose rescue analgesia and total nalbuphine intake postoperatively using Mann Whitney, unpaired student's t-test and chi-square between the two studied groups

Parameters	Group B (*n* = 30)	Group BD (*n* = 30)	*p*-value	MD or RR (95% CI)
Time for request of the initial dose rescue analgesia (minutes)	360(300–420)	690(525–795)	< 0.001* ^**U**^	330 (245: 415)
Number of patients needed nalbuphine consumption	17 (56.7%)	6 (20.0%)	0.004* ^**X2**^	2.83 (1.3: 6.19)
Total nalbuphine consumption (mg)	7.94 ± 2.54	5.83 ± 2.04	0.082^t^	2.11(0.92: 3.3)

Data are presented as mean ±SD, median (IQR) or frequency (%), *U* Mann Whitney test, *t* Unpaired student's t-test, *MD* Mean or median difference, *X*^2^ Chi-square, *RR* Relative risk, *CI* Confidence interval

*Significantly different as P-value<0.05

The VAS score was markedly decreased in group BD compared to group B at 6 and 12 h after surgery (p-value < 0.001). Nevertheless, no statistically notable distinction was documented between the two groups in VAS scores at the PACU, 1, 2, and 24 h postoperatively (P-value > 0.05), as shown in Table 3.

**Table 3:** Comparison of visual analogue score using Mann Whitney test between the two studied groups

	Group B(n=30)	Group BD(n=30)	p-value	MD (95% CI)
VAS at PACU	2 (2 - 3)	2 (2 - 2)	0.091^U^	0 (-1: 0)
VAS 1 hr	2 (2 - 3)	2 (2 - 2)	0.057^U^	0 (-1: 0)
VAS 2 hr	2 (2 - 3)	2 (2 - 2.75)	0.332^U^	0 (-1: 0)
VAS 6 hr	4 (4 - 4)	1 (1 - 2)	<0.001*^U^	-3 (-3: -2)
VAS 12 hr	4 (4 - 4)	1 (1 - 1.75)	<0.001*^U^	-3 (-3: -3)
VAS 24 hr	2 (2 - 3)	2 (2 - 3)	0.268^U^	0 (0: 0)

Data are presented as median (IQR), *VAS* Visual analogue score, *U* Mann Whitney test, *MD* Median difference, *CI* Confidence interval

*Significantly different as *P*-value<0.05

Data are presented as median (IQR), VAS: Visual analogue score; U: Mann Whitney test, MD: Median difference, CI: Confidence interval. *: Significantly different as P-value < 0.05.

When it came to the adverse events during the study (hypotension, bradycardia, postoperative nausea and vomiting and postoperative sedation), there was no statistically significant distinction between groups B and BD (p-value > 0.05), Respiratory depression and block-related complications didn’t occur in any patients in both groups as shown in Table 4.

**Table 4:** Comparison of adverse events using Fisher exact test between the two studied groups

Adverse events	Group B (*n* = 30)	Group BD (*n* = 30)	*p*-value	RR (95% CI)
Hypotension	4 (13.33%)	7 (23.33%)	0.506 ^FE^	0.57(0.19:1.75)
Bradycardia	2 (6.67%)	5 (16.67%)	0.424 ^FE^	0.4(0.08:1.9)
Postoperative nausea and vomiting	4 (13.3%)	2 (6.7%)	0.671^FE^	1.39 (0.74–2.60)
Postoperative sedation (Ramsey sedation score > 2)	0 (0%)	2 (6.7%)	0.492 ^FE^	---
Respiratory depression	0 (0%)	0 (0%)	NA	---
Block-related complications	0 (0%)	0 (0%)	NA	---

Data are presented as frequency (%), *NA* Not applicable, *FE* Fisher exact test, *RR* Relative risk, *CI* Confidence interval

## Discussion

According to the findings of the current study, adding dexmedetomidine to bupivacaine in US-guided ESPB for patients undergoing modified radical mastectomy was associated with better analgesia, which was apparent by lengthening the period required to demand the initial dose of rescue analgesia, reduction of VAS, and less consumption of postoperative nalbuphine in 24 h. But demographic data were insignificantly different in both groups. Perineural dexmedetomidine blocks the hyperpolarization-activated cation current, affecting the activity of peripheral nerves. Also, it can lead to vasoconstriction at the site of the injection, resulting in effect prolongation by delaying LA absorption. Moreover, it has analgesic properties [10].

The findings of our study were supported by another study, which assessed the function of ropivacaine plus dexmedetomidine in ESPB during video-assisted thoracoscopic lobectomy surgery. Their findings supported ours since they demonstrated that the dexmedetomidine group’s postoperative analgesic consumption was much less than that of the control group. Additionally, the dexmedetomidine group postponed the initial application of rescue analgesia [11].

Proceeding with our investigation, Wang Q. et al. [12] demonstrated that incorporating (0.5 µg/kg) of dexmedetomidine into ropivacaine 0.5% ]2 mL of normal saline (group R) with 28 mL of 0.5% ropivacaine or 0.5 µg/kg dexmedetomidine in 2 mL (group RD)[ in US-guided ESPB in thoracotomy for patients with esophageal cancer could successfully extend the period of post-surgical analgesia and decrease opioid use without raising the risk of side effects, We have achieved results with only half this concentration and a smaller volume.

Proceeding with our results, another study showed that adding dexmedetomidine to bupivacaine in high-thoracic ESPB for analgesia in shoulder arthroscopy showed lower VAS scores at 12 and 18 h postoperatively (p-value = 0.001 and 0.001, respectively) than ESPB with bupivacaine only and also prolonged the postoperative analgesia [13].

These findings are in harmony with the study of Manzoor [14], that showed that adding 30 ml of 0.25% dexmedetomidine to bupivacaine during Pectoralis Nerve Block (Pecs II) substantially extended the period of postoperative analgesia by approximately 40% when relative to using Bupivacaine alone (1024.0 ± 124.9 vs. 726.4 ± 155.3 min; *P* < 0.001), we used smaller volume than that study.

Similarly, Biçer et al. [15] performed a study involving 93 patients undergoing thoracotomy and demonstrated that the use of dexmedetomidine in a paravertebral block significantly improves postoperative pain management. Specifically, the administration of 20 mL of 0.5% bupivacaine combined with 1 mL of dexmedetomidine (100 µg) resulted in lower post-surgical pain scores and decreased morphine usage.

In agreement with our outcomes, Gad and El-Metwally [16] assessed the advantage of combining levobupivacaine 0.25% with dexmedetomidine (0.5 µg/kg) in an ultrasound-guided serratus plane block for MRM. According to their findings, adding dexmedetomidine to levobupivacaine resulted in a considerable reduction in the total amount of pethidine consumed after surgery and VAS at 8 and 12 h after the surgery. Additionally, the duration of sufficient analgesia was significantly extended.

In contrast to our trial, Packiasabapathy et al. [17] demonstrated that adding 1 µg/kg of dexmedetomidine to bupivacaine in femoral nerve block did not reduce morphine consumption significantly after total knee arthroplasty.

The discrepancy in findings may reflect a combination of factors. These include differences in local anesthetic volume and concentration, the dose of dexmedetomidine used, and the characteristics of the targeted neural structures. Fascial plane blocks such as the ESPB allow for wider multidermatomal spread and may affect both dorsal and ventral rami, potentially enhancing analgesic efficacy compared with more localized peripheral nerve blocks such as the femoral nerve block.

Additionally, the nature of postoperative pain may play a role. Femoral nerve blocks predominantly address somatic pain from the anterior thigh and knee, whereas thoracic and truncal fascial plane blocks may provide broader coverage, including deep somatic and, to some extent, visceral pain components. Variations in surgical procedures and pain profiles across studies may therefore influence the observed analgesic outcomes.

Although dexmedetomidine was administered perineurally, the possibility of systemic absorption should be acknowledged. Dexmedetomidine is known to exert dose-dependent systemic effects, including bradycardia and hypotension. However, in the present study, the low weight-adjusted dose (1 µg/kg), absence of clinically significant hemodynamic instability, and lack of increased sedation or respiratory depression suggest that systemic absorption, if present, was minimal and clinically insignificant.

These findings are consistent with previous studies reporting the safety of perineural dexmedetomidine in regional anesthesia when used within recommended dosing ranges.

Bao et al. [18] demonstrated that dexmedetomidine used as an adjuvant to local anesthetics prolonged analgesia without increasing clinically significant bradycardia, hypotension, or respiratory depression, suggesting minimal systemic impact at low doses.

Similarly, Chen et al. [19] reported that perineural dexmedetomidine provided effective analgesic prolongation with a favorable safety profile, and that observed hemodynamic effects were mild, transient, and not clinically significant.

There were limitations in our study, including a limited follow-up period (only 24 h), and our organizational population study was conducted in a single center; its findings might not apply to other groups. Additionally, in a few instances, the presence of postoperative edema or hematoma impacted the quality of ultrasound imaging. Cases with ASA Ⅲ or Ⅳ were excluded to avoid comorbid complications, this can affect the generalizability of the study to the broader population. The authors recommend future multi-center studies with larger sample size and inclusion of ASA Ⅲ and Ⅳ patients in the subsequent research.

## Conclusions

Our study suggests that adding dexmedetomidine to bupivacaine in ultrasound-guided ESPB for patients performing modified radical mastectomy will probably prolong postsurgical analgesia, reduce pain scores, improve hemodynamics, and decrease postoperative nalbuphine use.

## Supplementary Information

Supplementary Material 1.

Supplementary Material 2.

Supplementary Material 3.

Supplementary Material 4.

## Data Availability

All these data are available upon reasonable request from the corresponding author.

## References

[1] Albi-Feldzer A, Dureau S, Ghimouz A, Raft J, Soubirou J-L, Gayraud G, et al. Preoperative paravertebral block and chronic pain after breast cancer surgery: a double-blind randomized trial. Anesthesiol. 2021;135(6):1091–103. 10.1097/ALN.0000000000003989 34618889

[2] Kaur U, Shamshery C, Agarwal A, Prakash N, Valiveru RC, Mishra P. Evaluation of postoperative pain in patients undergoing modified radical mastectomy with pectoralis or serratus-intercostal fascial plane blocks. Korean J Anesthesiol. 2020;73(5):425–33. 32987492 10.4097/kja.20159 PMC7533170

[3] Wijayasinghe N, Andersen KG, Kehlet H. Neural Blockade for persistent pain after breast cancer surgery. Reg Anesth Pain Med. 2014;39(4):272–300. 24918332 10.1097/AAP.0000000000000101

[4] Forero M, Adhikary SD, Lopez H, Tsui C, Chin KJ. The erector spinae plane block: a novel analgesic technique in thoracic neuropathic pain. Reg Anesth Pain Med. 2016;41(5):621–7. 27501016 10.1097/AAP.0000000000000451

[5] Moharam SA, ElSharkawy MS, Almohasseb MA, Hamama K, Mahmoud MA, Abogabal MA. Ultrasound-guided external oblique intercostal plane block versus thoracic erector spinae block for post-thoracotomy pain: A randomised double-blinded non-inferior clinical study. Indian J Anaesth. 2025;69(8):809–15. 40800700 10.4103/ija.ija_3_25 PMC12338484

[6] Bao N, Shi K, Wu Y, He Y, Chen Z, Gao Y, et al. Dexmedetomidine prolongs the duration of local anesthetics when used as an adjuvant through both perineural and systemic mechanisms: a prospective randomized double-blinded trial. BMC Anesthesiol. 2022;22(1):176. 35672660 10.1186/s12871-022-01716-3 PMC9172023

[7] Chen Z, Liu Z, Feng C, Jin Y, Zhao X. Dexmedetomidine as an adjuvant in peripheral nerve block. Drug Des Devel Ther. 2023;17:1463–84. 10.2147/DDDT.S405294 PMC10200118 37220544

[8] Steverink JG, Piluso S, Malda J, Verlaan J-J. Comparison of in vitro and in vivo toxicity of bupivacaine in musculoskeletal applications. Front Pain Res. 2021;2:723883. 10.3389/fpain.2021.723883 PMC8915669 35295435

[9] Yousif SH, Zanfaly HI, Galal Eldin AM. Ultrasound-guided erector spinae block versus serratus anterior block for perioperative analgesia in patients undergoing modified radical mastectomy surgery. Res Opin Anaesth Intensive Care. 2024;11(4):512–32.

[10] Gao Z, Xiao Y, Wang Q, Li Y. Comparison of Dexmedetomidine and dexamethasone as adjuvant for ropivacaine in ultrasound-guided erector spinae plane block for video-assisted thoracoscopic lobectomy surgery: a randomized, double-blind, placebo-controlled trial. Ann Transl Med. 2019;7(2):668–78. 31930069 10.21037/atm.2019.10.74 PMC6944602

[11] Rao J, Gao Z, Qiu G, Gao P, Wang Q, Zhong W, et al. Nalbuphine and Dexmedetomidine as adjuvants to ropivacaine in ultrasound-guided erector spinae plane block for video-assisted thoracoscopic lobectomy surgery: A randomized, double-blind, placebo-controlled trial. Med (Baltim). 2021;100(32):269–362. 10.1097/MD.0000000000026962 PMC8360433 34397949

[12] Wang Q, Li H, Wei S, Zhang G, Ni C, Sun L, et al. Dexmedetomidine added to ropivacaine for ultrasound-guided erector spinae plane block prolongs analgesia duration and reduces perioperative opioid consumption after thoracotomy: A randomized, controlled clinical study. Clin J Pain. 2021;38(1):8–14. 34636753 10.1097/AJP.0000000000000992 PMC8635250

[13] Hamed MA, Fargaly OS, Abdelghaffar RA, Moussa MA, Algyar MF. The role of Dexmedetomidine as an adjuvant for high-thoracic erector spinae plane block for analgesia in shoulder arthroscopy; a randomized controlled study. BMC Anesthesiol. 2023;23(1):53–6. 36793000 10.1186/s12871-023-02014-2 PMC9930274

[14] Manzoor S, Taneja R, Sood N, Puri A, Kadayaprath G. Comparative study to assess the quality of analgesia of bupivacaine and bupivacaine with Dexmedetomidine in ultrasound-guided pectoral nerve block type I and II in breast surgeries. J Anaesthesiol Clin Pharmacol. 2018;34(2):227–31. 30104834 10.4103/joacp.JOACP_366_16 PMC6066886

[15] Biçer C, Ünalan EN, Aksu R, Önal Ö, Güneş I. [Addition of Dexmedetomidine to bupivacaine in ultrasonography-guided paravertebral Blockade potentiates postoperative pain relief among patients undergoing thoracotomy]. Braz J Anesthesiol. 2019;69(2):144–51. 30665671 10.1016/j.bjane.2018.12.004 PMC9391857

[16] Gad M, Elmetwally M. Efficacy of adding Dexmedetomidine as adjuvant with Levobupivacaine in ultrasound-guided serratus plane block for modified radical mastectomy surgery. Res Opin Anaesth Intensive Care. 2019;6(2):512–32.

[17] Packiasabapathy SK, Kashyap L, Arora MK, Batra RK, Mohan VK, Prasad G, et al. Effect of Dexmedetomidine as an adjuvant to bupivacaine in femoral nerve block for perioperative analgesia in patients undergoing total knee replacement arthroplasty: A dose-response study. Saudi J Anaesth. 2017;11(3):293–8. 28757829 10.4103/sja.SJA_624_16 PMC5516491

[18] Bao N, Shi K, Wu Y, He Y, Chen Z, Gao Y, et al. Dexmedetomidine prolongs the duration of local anesthetics when used as an adjuvant through both perineural and systemic mechanisms: a prospective randomized double-blinded trial. BMC Anesthesiol. 2022;22(1):176–87. 35672660 10.1186/s12871-022-01716-3 PMC9172023

[19] Chen Z, Liu Z, Feng C, Jin Y, Zhao X. Dexmedetomidine as an adjuvant in peripheral nerve block. Drug Des Devel Ther. 2023;17(8):1463–84. 37220544 10.2147/DDDT.S405294 PMC10200118

