# Supplementary material for: Effect of adding dexmedetomidine as an adjuvant to bupivacaine in ultrasound-guided erector spinae plane block for postoperative pain management following modified radical mastectomy: a randomized controlled trial
Source: BMC Surg. 2026 Mar 4;26:251. doi: 10.1186/s12893-026-03557-0 (PMC13064333; doi:10.1186/s12893-026-03557-0)
Supplement: Supplementary file 1 — Supplementary Material 1. [file 12893_2026_3557_MOESM1_ESM.docx]

**PROTOCOL OF A THESIS FOR PARTIAL FULFILMENT OF
M.D IN ANETHESIOLOGY**

**Title of the Protocol:** Effect of adding dexmedetomidine as an adjuvant to bupivacaine in ultrasound-guided erector spinae plane block for post-modified radical mastectomy pain management

**Postgraduate Student: Abanoub Mouris Feltaoos habib**

**DEGREE:** M.B,B.Ch, M.Sc, Ain shams university.

**DIRECTOR: Prof. Hatem Said Abdelhamed**

**Academic Position:** Professor

**Department:** Professor of Anesthesia, Intensive care and Pain Management, Faculty of Medicine - Ain Shams University

**Co-DIRECTOR: Prof. Karim Youssef Kamal**

**Academic Position**: Professor

**Department:** Professor of Anesthesia, Intensive care and Pain Management, Faculty of Medicine - Ain Shams University

**Co-DIRECTOR: Prof. Ashraf Elsayed Elagamy**

**Academic Position:** Professor

**Department:** Professor of Anesthesia, Intensive care and Pain Management, Faculty of Medicine - Ain Shams University

**Co-DIRECTOR: Dr. Ahmed Mostafa Abdulmageed**

**Academic Position:** Lecturer

**Department:** Lecturer of Anesthesia, Intensive care and Pain Management, Faculty of Medicine - Ain Shams University

**Faculty of Medicine**

**Ain Shams University**

**2023**

| **What is already known on this subject? AND**  **What does this study add?** |
| --- |
| Peripheral nerve blocks are effective adjuvant options for pain management in breast surgeries. The use of Erector spinae plane block (ESPB) has been proven to be very effective at controlling pain and minimizing narcotic consumption after modified radical mastectomy surgeries.  Many adjuvants to local anaesthetics were used to improve the duration and intensity of the peripheral nerve block. We will study the effect of adding dexmedetomidine to bupivacaine in ESPB after modified radical mastectomy surgeries. |

| **1.INTRODUCTION/ REVIEW** |
| --- |
| Breast cancer is one of the most important medical problems in the female gender, since among every eight women, one will suffer from breast cancer during her lifetime ***(Fizgerald, 2015).***  Acute postoperative pain is very common after breast surgeries which requires adequate pain management. Different peripheral nerve blocks such as paravertebral block were used as an analgesia for breast surgeries ***(Vila et al., 2007).***  ESPB is a newer interfascial plane block first described by Forero et al, who used it for treating thoracic neuropathic pain by injecting a local anesthetic deep into the erector spinae muscle at the level of T5. Moreover, ESPB is a reasonable method to administer, with clearly identifiable sonographic landmarks and LA needle insertion and injection locations. ESPB has been applied in thoracic and abdominal surgeries, with high success rates providing both visceral and somatic analgesia ***(Forero and Rajarathinam , 2017).*** Later studies have shown that ESPB can provide effective analgesia in breast surgeries ***(Singh and Chowdary, 2018)****.*  ESPB achieved by injecting the local anesthetics locally in the deep erector spinae muscle surface, as a part of multimodal analgesia. Given that erector spinae muscles anatomically situate along the thoracolumbar spine, ESPB promotes an extensive craniocaudal spread ***(El-Boghdadly and Pawa, 2017).***  Ultrasound is a non-invasive visualization technology that helps capture the anatomical structure of target tissues; it can help guide the direction and depth of anesthesia puncture needles, thus reducing the risk of complications ***(Chang et al., 2020).***  Adjuvants to local anasthetics, such as opioids, alpha 2 agonists, magnesium and dexamethasone may improve the duration and intensity of peripheral nerve blocks effect ***(Swain et al., 2017).*** |

| **2.AIM / OBJECTIVES** |
| --- |
| The aim of the study is to assess the effect of adding dexamedetomidine to bupivacaine in US guided erector spinae plane block for modified radical mastectomy surgeries , Using visual analogue score and post-operative narcotics consumption. |

| **3.METHODOLOGY:**  **Patients and Methods/ Subjects and Methods/ Material and Methods** |
| --- |
| - **Type of study:** Prospective double-blinded, randomized controlled clinical pilot study after approval was obtained from research ethics committee of anaesthesia and intensive care department, Ain Sahms University, Cairo, Egypt (FMASU MD145/2023) - **Study Setting**: Ain shams University Hospital, Cairo, Egypt. - **Study Period**: Expected for one year starting from september 2023. - **Study population:** - **Inclusion Criteria:** - Age:30-65years - Physical status: ASA I,II patients after taking written and informed consent. - BMI<35 - **Exclusion criteria:**   Age:<30 and >65 years   - Refusal of procedure or participation in the study by the patient. - Physical status: ASA III or above. - History of allergy to the study drug. - BMI>35 - Bleeding disorders and coagulopathy. - Psychiatric illness that may interfere with the study. - **Sampling Method**: Random. - **Sampling Size: 60 patients** - Based on the results of Elshal et al 2021 , with the median time to request rescue analgesia in patients without dexamedetomidine 3 hours compared to 6 hours in patients with dexamedetomidine , alpha error 5% and power of study 80%, the required sample is 60 patients 30 in each group . the program for sample size calculation is STATA 10. - **Ethical considerations**: Approval of the research ethical committee of faculty of medicine, Ain Shams University will be obtained and informed consent will be taken from all participants.   **Study Procedure:**  **Pre-operative Settings:**  Preoperative assessment of all patients will be done which includes history taking, number of fasting hours, clinical examination and standard laboratory testing including Complete blood picture, Bleeding time, Prothrombine time and partial thromboplastine time, AST, ALT, Urea, Creatinine and viral markers. The procedure will be explained to the patients and their consent will be taken. All patients will be familiar with the use of pain score (visual analogue scale score), Identifying 0 as no pain and 10 as the worst pain.  **Study medications will be prepared in two sets:**   1. 10 ml Bupivacaine 0.5% added to 10 ml normal saline. 2. 10 ml Bupivacaine 0.5% added to 1 ml dexmedetomidine (1 μg/kg) + 9ml normal saline.   Two sets will be prepared in a sterile syringes and will be given to the investigator who will be blinded to identify the drug.  **Groups:**  **Patients will be randomly divided in to 2 groups:**   - **Group B:** consists of patients receiving ESPB with Bupivacaine only. - **Group BD:** consists of patients receiving ESPB using bupivacaine plus dexmedetomidine.   **Intraoperative settings:**  On arrival of the patient to the operating room a 20 gauge iv access will be inserted at the contralateral side of the surgery and anti-stress ulcer measures: intravenous pantoprazole 40mg will be given, lactated ringer solution will be infused at a rate 10ml/hr and the patient will receive titration of 1 mg midazolam as a sedation. ASA standard Monitors for heart rate, non invasive blood pressure, electrocardiogram and pulse oximetry will be applied to monitor the perioperative vital signs of the patients, Induction of general anaesthesia will be performed using iv propofol (2mg/kg), fentanyl (1ug/kg) and atracrurium (0.5mg/kg) .After tracheal intubation, anesthesia will be maintained using isoflurane MAC 1.2% and atracrurium (0.1mg/kg) as indicated by nerve stimulator.  At the end of surgery, the patient will be placed in the lateral position. Skin sterilization will be done first then a high frequency linear probe will be placed in a longitudinal parasagittal plane approximately 3 cm from midline. After identifying the erector spinae muscle superficial to the tip of T5 transverse process, An echogenic needle of 22G will be inserted with an in-plane approach in a cranio-caudal manner till it contacts with the tip of transverse process. After slight retraction of the needle the local anaesthetics will be injected after negative aspiration to avoid intravascular injection and the spread of the drug will be observed in tissue plane under ultrasound imaging  **Post-operative settings:**  Reversal of muscle relaxants will be achieved by atropine 0.02 mg/kg and neostigmine 0.05mg/kg at the end of surgery and Extubation will be done after complete recovery of the airway reflexes.  Then patients will be transferred to the post anesthesia care unit (PACU) and will be observed for one hour. A modified aldrete score>9 will be required for discharge from the PACU to the ward.  VAS (visual analog score), mean arterial pressure and heart rate will be recorded at (0,1,2,6,12 and 24 hours) post operatively. Rescue analgesia (on demand analgesia given to control breakthrough pain) in form of 5 mg nalbuphine (0.1ml/kg) will be given whenever VAS score > 4 and 1gm paracetamol will be given every 8 hours.  **Primary and secondary outcomes:**  The primary outcome is the first time to request rescue analgesia.  The secondary outcome will be to measure the VAS scores at different points in the study and the total opioids consumbtion over 24 hours after surgery.   - **Statistical analysis**:   The collected data will be revised, coded and introduced to a PC using Statistical Package for Social Science (SPSS) program.  Data will be presented as mean and standard deviation(+-SD) for quantitative parametric data, median and range for quantitative non parametric data and as numbers and percentage for qualitative data. Suitable analysis will be done according to the type of data obtained. P value<0.05 will be considered significant. |

| **4.REFERENCES:** |
| --- |
| 1. **Chang, P. H., Chen, Y. J., Chang, K. V., Wu, W. T., & Özçakar, L. (2020). Ultrasound measurements of superficial and deep masticatory muscles in various postures: reliability and influencers. Scientific reports, 10(1), 14357.** 2. **El-Boghdadly, K., & Pawa, A. (2017). The erector spinae plane block: plane and simple. Anaesthesia, 72(4), 434–438.** 3. **Fitzgerald S. P. (2015). Breast-Cancer Screening--Viewpoint of the IARC Working Group. The New England journal of medicine, 373(15), 1479.** 4. **Forero, M., Rajarathinam, M., Adhikary, S., & Chin, K. J. (2017). Continuous Erector Spinae Plane Block for Rescue Analgesia in Thoracotomy After Epidural Failure: A Case Report. A & A case reports, 8(10), 254–256.** 5. **Singh, S., & Chowdhary, N. K. (2018). Erector spinae plane block an effective block for post-operative analgesia in modified radical mastectomy. Indian journal of anaesthesia, 62(2), 148–150.** 6. **Swain, A., Nag, D. S., Sahu, S., & Samaddar, D. P. (2017). Adjuvants to local anesthetics: Current understanding and future trends. World journal of clinical cases, 5(8), 307–323.** 7. **Vila, H., Jr, Liu, J., & Kavasmaneck, D. (2007). Paravertebral block: new benefits from an old procedure. Current opinion in anaesthesiology, 20(4), 316–318.** |
