# Supplementary material for: Effect of adding dexmedetomidine as an adjuvant to bupivacaine in ultrasound-guided erector spinae plane block for postoperative pain management following modified radical mastectomy: a randomized controlled trial
Source: BMC Surg. 2026 Mar 4;26:251. doi: 10.1186/s12893-026-03557-0 (PMC13064333; doi:10.1186/s12893-026-03557-0)
Supplement: Supplementary file 3 — Supplementary Material 3. [file 12893_2026_3557_MOESM3_ESM.docx]

**Dexmedetomidine**

Dexmedetomidine is a highly selective alpha-2 adrenergic receptor agonist, is of strong sedative, analgesic, anesthetic sparing effects when used in general anesthesia.^1^ In the meantime, it can also be used as a perineural adjuvant to facilitate better anesthesia and analgesia. prolong the duration of sensory block, motor block and analgesia, and accelerate the time to onset of sensory and motor block.^1 2^

**Mechanism of action of dexmedetomidine**:

The hypothesized mechanisms of dexmedetomidine administration in the peripheral nerve block are as follows: dexmedetomidine inhibits the function of sodium channels and neuronal potassium current ^3^ and blocks the hyerpolarization-activated cyclic nucleotide-gated channels, resulting in the enhancement of activity-dependent hyperpolarization ^4^ and leading to the inhibition of substance P release in the nociceptive pathway at the dorsal root neuron.^5^

**Comparative Studies of dexmedetomidine with Other Drugs**:

1) Dexmedetomidine vs clonidine

Compare clonidine and dexmedetomidine in supraclavicular Brachial Plexus Block (BPB) When added to local anesthetic in the supraclavicular BPB, dexmedetomidine enhanced the duration of sensory and motor block and analgesia. The time for rescue analgesia was prolonged in patients receiving dexmedetomidine. It also enhanced the quality of the block as compared with clonidine.^6^

This suggests that dexmedetomidine may have a better analgesic effect and longer analgesic time as an adjuvant.

2) Dexmedetomidine vs nalbuphine

Compare dexmedetomidine and nalbuphine in Thoracic Paravertebral Block (TPVB) in breast cancer surgeries. Adding nalbuphine to TPVB improved the block’s quality and decreased postoperative analgesic requirements. Adding dexmedetomidine to bupivacaine increased the time to the first analgesic request and more sedation than bupivacaine and bupivacaine with nalbuphine.^7^

Although nalbuphine has more advantages in analgesic quality, dexmedetomidine can prolong the analgesic time and provide stronger sedation.

3) Dexmedetomidine vs dexamethasone

Compare dexamethasone and dexmedetomidine for the infraclavicular BPB in patients undergoing forearm fracture surgery there was no significant difference in postoperative pain intensity between dexamethasone and dexmedetomidine groups, although dexmedetomidine demonstrated a longer sensory block duration in comparison with dexamethasone as a lidocaine adjuvant in infraclavicular BPB.^8^

4) Dexmedetomidine vs magnesium sulfate

Compare dexmedetomidine and MgSO4 in supraclavicular BPB

dexmedetomidine provides earlier onset of the sensory and motor block and a prolonged duration of sensory and motor blocks, the duration of analgesia is longer, and postoperative rescue analgesia is less compared to patients receiving MgSO4.^9^

5) Dexmedetomidine vs fentanyl

Compare dexmedetomidine and fentanyl under ultrasound guidance in supraclavicular BPB, dexmedetomidine prolongs the duration of sensory and motor block and postoperative analgesia as compared to fentanyl when used as an adjuvant to ropivacaine in supraclavicular BPB and is not associated with any major adverse events.^10^

**Dose of dexmedetomidine in Peripheral Nerve Blocks:**

The optimal dose of dexmedetomidine as an adjuvant in peripheral nerve block depends on the dose, the block location, the concentration and dose of the local anesthetic drug, surgical method, population, and other factors. Additionally, the choice of dose varies according to the investigator’s purpose.

Many studies have established that the maximum safe dose of dexmedetomidine is 2 μg/kg, and the duration of continuous analgesia is the longest, but the incidence of hypotension is higher in 1.5–2 μg/kg dexmedetomidine.^11^ Thus, a dose of dexmedetomidine of 1 μg/kg provides an optimal balance between adequate postoperative analgesia and the adverse effects of peripheral nerve block.^12^

**Neurotoxicity of dexmedetomidine:**

Multiple studies have examined the risk of neurotoxicity in perineurally administered dexmedetomidine. Schnabel et al, for example, summarized that the injection of rat sciatic nerves with dexmedetomidine in combination with either ropivacaine or bupivacaine did not show significant changes within the nerve compared with local LAs alone. Furthermore, adding dexmedetomidine to LAs might have a protective effect on reducing the inflammatory effects of LA by regulating mast cell degranulation.^13^

It is suggested that the neuroprotective effect of dexmedetomidine makes it suitable for peripheral nerve block as a combined local anesthetic.

**References**

1. **Bajwa, S., & Kulshrestha, A. (2013). Dexmedetomidine: an adjuvant making large inroads into clinical practice. *Annals of medical and health sciences research*, *3*(4), 475–483.**
2. **Bhana, N., Goa, K. L., & McClellan, K. J. (2000). Dexmedetomidine. Drugs, 59(2), 263–270.**
3. **Gu, X. Y., Liu, B. L., Zang, K. K., Yang, L., Xu, H., Pan, H. L., Zhao, Z. Q., & Zhang, Y. Q. (2015). Dexmedetomidine inhibits Tetrodotoxin-resistant Nav1.8 sodium channel activity through Gi/o-dependent pathway in rat dorsal root ganglion neurons. Molecular brain, 8, 15.**
4. **Brummett, C. M., Hong, E. K., Janda, A. M., Amodeo, F. S., & Lydic, R. (2011). Perineural dexmedetomidine added to ropivacaine for sciatic nerve block in rats prolongs the duration of analgesia by blocking the hyperpolarization-activated cation current. Anesthesiology, 115(4), 836–843.**
5. **Guo, T. Z., Jiang, J. Y., Buttermann, A. E., & Maze, M. (1996). Dexmedetomidine injection into the locus ceruleus produces antinociception. Anesthesiology, 84(4), 873–881.**
6. **Swami, S. S., Keniya, V. M., Ladi, S. D., & Rao, R. (2012). Comparison of dexmedetomidine and clonidine (α2 agonist drugs) as an adjuvant to local anaesthesia in supraclavicular brachial plexus block: A randomised double-blind prospective study. Indian journal of anaesthesia, 56(3), 243–249.**
7. **Omar Mostafa, M., Makram Botros, J., & Sayed Khaleel, A. M. (2018). Effect of Dexmedetomidine Versus Nalbuphine as an Adjuvant on Paravertebral Block to Manage Postoperative Pain After Mastectomies. Anesthesiology and pain medicine, 8(2), e13308.**
8. **Yaghoobi, S., Shahamat, H., Alizadeh, A., & Khezri, M. B. (2019). Comparing Postoperative Analgesic Effect of Dexmedetomidine or Dexamethasone Added to Lidocaine Through Infraclavicular Block in Forearm Surgery. The Clinical journal of pain, 35(9), 766–771.**
9. **Shukla, U., Singh, D., Yadav, J. B. S., & Azad, M. S. (2020). Dexmedetomidine and Magnesium Sulfate as Adjuvant to 0.5% Ropivacaine in Supraclavicular Brachial Plexus Block: A Comparative Evaluation. Anesthesia, essays and researches, 14(4), 572–577.**
10. **Dharmarao, P. S., & Holyachi, R. (2018). Comparative Study of the Efficacy of Dexmedetomidine and Fentanyl as Adjuvants to Ropivacaine in Ultrasound-Guided Supraclavicular Brachial Plexus Block. Turkish journal of anaesthesiology and reanimation, 46(3), 208–213.**
11. **Vorobeichik, L., Brull, R., & Abdallah, F. W. (2017). Evidence basis for using perineural dexmedetomidine to enhance the quality of brachial plexus nerve blocks: a systematic review and meta-analysis of randomized controlled trials. British journal of anaesthesia, 118(2), 167–181.**
12. **Reddy, B. S., Gaude, Y. K., Vaidya, S., Kini, G. K., Budania, L. S., & Eeshwar, M. V. (2021). Effect of dexmedetomidine on characteristics of ultrasound-guided supraclavicular brachial plexus block with levobupivacaine-A prospective double-blind randomized controlled trial. Journal of anaesthesiology, clinical pharmacology, 37(3), 371–377.**
13. **Schnabel, A., Reichl, S. U., Weibel, S., Kranke, P., Zahn, P. K., Pogatzki-Zahn, E. M., & Meyer-Frießem, C. H. (2018). Efficacy and safety of dexmedetomidine in peripheral nerve blocks: A meta-analysis and trial sequential analysis. European journal of anaesthesiology, 35(10), 745–758.**
